# In the heart of cardiac stromal senescence

**DOI:** 10.18632/aging.102806

**Published:** 2020-01-27

**Authors:** Victorine Douin-Echinard, Lise Lefevre, Angelo Parini

**Affiliations:** 1 Institute of Cardiovascular and Metabolic Diseases, Inserm UMR1048, Toulouse, France; 2 Paul Sabatier University, Toulouse, France; 3 University Hospital of Toulouse, Toulouse, France

**Keywords:** heart, senescence, mesenchymal stromal cells, inflammaging, IL-1ß

At the cellular level, instead of progressive loss of function per se, aging is now perceived as a loss of cellular flexibility avoiding adaptive cellular processes such as switches in cellular metabolism and differentiation pathways in response to infection, ischemia, or metabolic stresses. This could result from cellular reprogramming to cope with damages caused by repetitive low grade extrinsic or intrinsic stresses. For now, organ specific gradation of biological aging is still challenging and relies on the better understanding of the biological processes altering organ homeostasis with aging.

In a recent study, we have demonstrated that physiological aging induces senescence of cardiac mesenchymal stromal cells (cMSCs) and, through senescence associated secretory phenotype (SASP) acquisition and differentiation bias, participates in modifications of the composition of cardiac microenvironment in aged C57BL/6JRj mice [2]. These results are in agreement with a previous study reporting increased frequencies of senescent human cardiac progenitor cells isolated from the atria of aged patients with cardiovascular diseases compared to middle-age patients [3].

**Figure 1:**
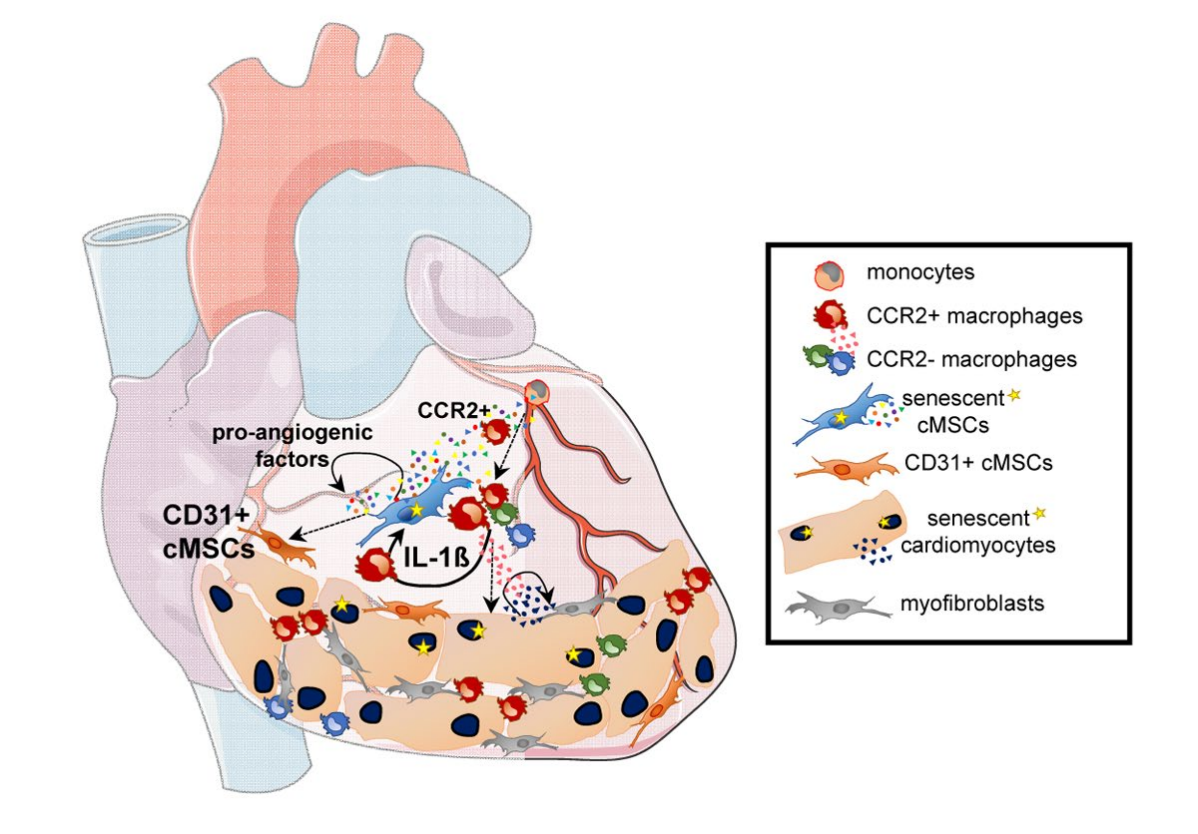
**Cross-talk evolution between cardiomyocytes and cardiac stromal cell subsets during aging.** During aging, cardiomyocytes and cardiac mesenchymal stromal cells (cMSC) acquire specific senescence associated secretory phenotypes (SASP) which promote age-related changes in the heart. Pro-angiogenic factors expressed by aged cMSCs increased frequencies of non-classical CD31+ cMSCs expressing endothelial related genes. SASP related pro-inflammatory mediators from cMSCs promote recruitment of monocyte-derived CCR2+ macrophages, with IL-1ß production re-enforcing cMSC senescence, cardiomyocyte hypertrophy and altered contractility. Concomitant acquisition of a non-conventional SASP by aged cardiomyocytes stimulates cardiomyocyte hypertrophy and myofibroblast activation.

We focused our analysis on cMSCs isolated from heart ventricles that play diverse regulatory roles in the microenvironment by their secretome and their differentiation potential in the vascular lineage [2]. Analysis of native cMSCs was performed by cell sorting by flow cytometry based on the co-expression of sca-1 and PDGFRα (S+P+) and lack of CD31 and CD45. Aged cMSCs recruit monocytes thanks to CCR2 activation in chemotaxis assay which was congruent with the higher proportion of activated cardiac CCR2+ macrophages in aged mice (Figure 1). Modification of the macrophage pool in the aging heart resulted in production of inflammatory cytokines such as IL-1ß (Figure 1). Interestingly, aged cMSCs expressed several IL-1ß responsive genes and higher levels of IL-1R1 compared to young so we hypothesized that functional changes of cMSCs could be due to modifications of cardiac macrophage pool and their secretions in the cardiac stroma of aged mice [2].

Accordingly, IL-1ß but not IFN-ß treatment *in vitro*, induces senescence of young cMSCs, increases expression of CCR2 ligands (*Ccl2*, *Ccl8*) and monocyte chemotaxis [2]. Hence, IL‐1ß derived from CCR2+ macrophages mediates paracrine senescence of cMSCs, re-enforcing a deleterious amplification loop by which senescent cMSCs could potentiate CCR2-dependent monocyte chemoattraction and increase frequencies of cardiac CCR2+ macrophages (Figure 1).

Recently, S+P+ stromal cells producing IL-33 have been identified in perivascular niches, in various tissues, where they control ILC2 and regulatory T cell activation [4], such as in the white adipose tissue (WAT) [5]. Single-cell transcriptomic analysis of WAT S+P+ identified 5 distinct sub-populations with specific tissue location and functions, such as adipogenesis or IL-33 production [5]. Evolution of such niches in the heart and the pericardial adipose tissue during aging will be of strong interest to better resolve the age-related changes of cardiac microenvironment and parenchymal function.

We then asked if cMSC aging could also directly impact cardiac microenvironment homeostasis by modifying frequencies of cMSC subsets with committed vascular differentiation potentials. Aged CD90+ cMSCs have higher potential to acquire endothelial markers *in vitro* and have strong similarities with endothelial vascular progenitor cells (EVPs), expressing CD90 and *Pdgfra* mRNA [6]. Unsupervised multidimensional flow cytometry analysis revealed that frequency of the classical CD90+ CD31- cMSCs decreases with aging while, concomitantly, a new CD90+ cMSC subset emerges (Figure 1), acquiring intermediate levels of CD31 and endothelial related genes [2].

Farbehi et al*.* have shown, by single-cell expression profiling, the diversity of cardiac stromal cells with at least 8 different subsets of fibroblasts in healthy young mice, and their dynamics in response to myocardial injury [7]. Based on comparative transcriptomic expression between fibroblast subsets, the CD90+ S+P+ cells were defined as the self-renewing population due to their highest stem/progenitor cell marker expression and clonogenic activity. Interestingly, a rare so-called “hybrid population” expressing PDGFR-α, CD31 and genes of the endothelial cell lineage was also described at steady state in young mice [7], resembling the CD31+ cMSC population we identified, up-regulated with aging [2].

Our hypothesis is that CD90+ cMSCs would give rise to CD31+ cMSCs, representing endothelial progenitors that are unable to fully differentiate towards mature endothelial cells in aged mice and which, accumulating with aging, could have impaired functions such as forming unconnected capillary-like structures or leaky vessels.

In the heart, aging also drives senescence of parenchymal cells (Figure 1) which is initiated by persistent telomere-associated foci and DDR activation and which concurs with the acquisition of a non-conventional SASP promoting cardiomyocyte hypertrophy and myofibroblast activation [8]. Furthermore, senescence of human cardiac progenitors and release of SASP related pro-inflammatory factors decreased their efficacy for cell therapy in an experimental mouse model of myocardial infarction [3]. Ablation of senescent p16 positive cells by gene suicide strategy or by senolytic treatments in mice, normalized cardiomyocyte mean size and reduced fibrosis demonstrating the deleterious role of senescent cells in cardiac aging [3,8].

Altogether, these results strongly suggest that the pathophysiological mechanisms leading to organ dysfunction should be specific to the elderly compared to the young leading to diseases with shared cardinal clinical symptoms but differing in their molecular initiation steps.

This hypothesis has strong repercussions for innovative research for the development of therapeutics dedicated to treat pathologies such as cardiovascular diseases in individuals with high cellular biological age scores.
